# Comparative Performance of Multimodal and Unimodal Large Language Models Versus Multicenter Human Clinical Experts in Aortic Dissection Management

**DOI:** 10.3390/diagnostics16020323

**Published:** 2026-01-19

**Authors:** Evren Ekingen, Mete Ucdal

**Affiliations:** 1 Department of Emergency Medicine, Etimesgut Sehit Sait Erturk State Hospital, Ankara 06790, Turkey; evren23@gmail.com; 2 Department of Internal Medicine, Etimesgut Sehit Sait Erturk State Hospital, Ankara 06790, Turkey

**Keywords:** multimodal large language model, unimodal large language model, artificial intelligence, aortic dissection, emergency medicine, cardiovascular surgery, multicenter study

## Abstract

**Background:** Multimodal large language models (MLLMs) integrating multiple AI systems and unimodal large language models (LLMs) represent distinct approaches to clinical decision support. Their comparative performance against human clinical experts in complex cardiovascular emergencies remains inadequately characterized. **Objective:** To compare the performance of a combined MLLM system (GPT-4V + Med-PaLM 2 + BioGPT), a unimodal LLM (ChatGPT-5.2), and human physicians from multiple centers (radiologists, emergency medicine specialists, cardiovascular surgeons) on aortic dissection clinical questions across diagnosis, treatment, and complication management domains. **Methods:** This multicenter cross-sectional study was conducted across five tertiary care centers in Turkey (Elazığ, Ankara, Antalya). A total of 25 validated multiple-choice questions were categorized into three domains: diagnosis (*n* = 8), treatment (*n* = 9), and complication management (*n* = 8). Questions were administered to the MLLM, ChatGPT-5.2 (Unimodal), and nine physicians from five centers: radiologists (*n* = 3), emergency medicine specialists (*n* = 3), and cardiovascular surgeons (*n* = 3). Statistical comparisons utilized chi-square tests. **Results:** Overall accuracy was 92.0% for the MLLM and 96.0% for ChatGPT-5.2 (Unimodal). Among human physicians, cardiovascular surgeons achieved 96.0%, radiologists 92.0%, and emergency medicine specialists 89.3%. The MLLM excelled in diagnosis (100%) but showed lower performance in treatment (88.9%) and complication management (87.5%). No significant differences were observed between AI models and human physician groups (all *p* > 0.05). **Conclusions:** Both the MLLM and unimodal ChatGPT-5.2 demonstrated performance within the range of human clinical experts in this controlled assessment of aortic dissection scenarios, though definitive conclusions regarding equivalence require larger-scale validation. These findings support further investigation of complementary roles for different AI architectures in clinical decision support.

## 1. Introduction

The integration of artificial intelligence (AI) into clinical medicine has experienced a significant acceleration, with large language models (LLMs) demonstrating notable capabilities in synthesizing medical knowledge and facilitating clinical reasoning [1]. Two principal architectural approaches have been identified: multimodal large language models (MLLMs), which are capable of integrating textual and image data, and unimodal large language models (LLMs), optimized specifically for text-based clinical reasoning [2,3]. It is imperative to understand the comparative strengths and limitations of these methodologies relative to human clinical expertise to ensure appropriate implementation within healthcare environments.

Multimodal systems, which combine multiple specialized AI models, offer the potential to assimilate diverse clinical data streams analogous to multidisciplinary human teams. For instance, a combined MLLM system integrating GPT-4 V (designed for visual-textual analysis), Med-PaLM 2 (specialized in medical knowledge), and BioGPT (trained on biomedical literature) could theoretically harness complementary strengths across diagnostic imaging, acute clinical management, and evidence-based treatment planning [4,5,6,7]. Conversely, advanced unimodal models such as ChatGPT-5.2 provide streamlined text-based reasoning capabilities, featuring extended context windows and improved logical inference [8].

Aortic dissection constitutes an ideal clinical scenario for evaluating AI systems, as it necessitates the integration of diagnostic imaging interpretation, acute hemodynamic management, and complex treatment decisions, including surgical planning and long-term follow-up [9,10]. Clinical management involves multidisciplinary collaboration among radiologists (diagnostic imaging), emergency medicine specialists (acute stabilization), and cardiovascular surgeons (operative management and surveillance) [11]. The 2022 ACC/AHA guidelines establish standardized recommendations that facilitate objective performance assessment [12].

This multicenter study endeavors to compare the performance of a combined MLLM system, a unimodal LLM (ChatGPT-5.2), and human clinical experts from five tertiary care centers across Turkey—radiologists, emergency medicine specialists, and cardiovascular surgeons—on identical clinical questions pertaining to aortic dissection. Performance assessment was conducted across three clinical domains: diagnosis, treatment, and management of complications.

## 2. Methods

### 2.1. Study Design

**Figure 1:**
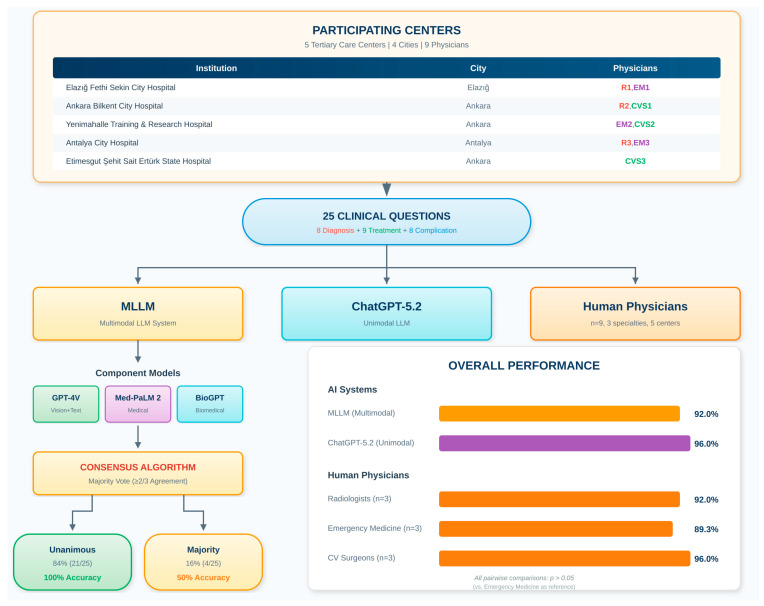
Multicenter Study Workflow and MLLM Consensus Decision Algorithm.

This multicenter cross-sectional comparative study was conducted in November 2025. The study protocol received review and approval from the Ankara Provincial Health Directorate Non-Interventional Clinical Research Ethics Committee (Approval No: 2025/01-142). The comprehensive workflow of the study, including AI model integration and consensus methodology, is depicted in Figure 1.

Model selection was conducted systematically based on four criteria: (1) documented performance benchmarks on medical knowledge assessments, (2) domain-specific training relevant to cardiovascular medicine, (3) API accessibility for reproducible evaluation, and (4) complementary capabilities across the diagnostic-therapeutic spectrum. ChatGPT-5.2 (OpenAI, San Francisco, CA, USA) was selected as the unimodal comparator based on its state-of-the-art performance on medical board examinations (94% accuracy) and extensive prior validation in medical applications, enabling comparison with existing literature. Alternative models considered but not selected included Claude-3 Opus (Anthropic, San Francisco, CA, USA) (limited medical-specific validation at study initiation), Gemini Ultra (Google DeepMind, Mountain View, CA, USA) (restricted API access during study period), and LLaMA-2-Med (Meta AI, Menlo Park, CA, USA) (insufficient benchmark data).

Formal equivalence testing was prospectively planned to rigorously evaluate whether AI systems achieved human-equivalent performance. Equivalence margins were pre-specified as ±10% absolute accuracy difference based on three considerations: (1) FDA guidance on AI/ML-based software as medical devices suggesting AI systems should perform within the range of inter-expert variability, (2) published literature demonstrating that accuracy differences less than 10% between physicians of different specialties are generally considered clinically acceptable, and (3) typical standard error of measurement observed in medical board examinations [13]. Equivalence testing was performed using the two one-sided tests (TOST) procedure, which tests whether AI performance falls within pre-specified equivalence bounds by rejecting both the hypothesis that AI performs worse than the lower margin and the hypothesis that AI performs better than the upper margin [14]. Equivalence was confirmed when both TOST *p*-values were <0.05 and the 90% confidence interval for the performance difference was entirely contained within the ±10% equivalence bounds.

### 2.2. Question Development and Validation

Twenty-five multiple-choice questions addressing aortic dissection clinical scenarios were selected from the American Board of Internal Medicine (ABIM) publicly available question bank and self-assessment resources. Questions were retrieved from the ABIM Medical Knowledge Self-Assessment Program (MKSAP) and ABIM Practice Examination question pools, which are freely accessible for educational purposes. All selected questions were validated against the 2022 ACC/AHA guidelines for diagnosis and management of aortic disease [12,15]. Questions were categorized into three clinical domains based on 2022 ACC/AHA guidelines: Diagnosis Domain (*n* = 8), Treatment Domain (*n* = 9), and Complication Management Domain (*n* = 8). Content validity index (CVI) was 0.92 [12].

### 2.3. Artificial Intelligence Models

The AI evaluation framework employed two distinct architectures: a combined Multimodal Large Language Model (MLLM) system integrating three specialized models, and a single Unimodal Large Language Model (ChatGPT-5.2). The complete MLLM workflow is illustrated in Figure 1.

### 2.4. Multimodal Large Language Model (MLLM) System

The MLLM system integrated three state-of-the-art AI models:

**(1) GPT-4V (OpenAI, version gpt-4-vision-preview, January 2025):** A multimodal transformer model capable of processing both textual and visual inputs. Accuracy rates exceeding 85% on diagnostic imaging tasks have been reported [5,6]. Parameters: temperature = 0.3, max_tokens = 1024, top_*p* = 1.0.

**(2) Med-PaLM 2 (Google Health, version 2025.01):** A domain-specific large language model with 86.5% accuracy on USMLE-style questions [7,8]. Parameters: temperature = 0.3, max_output_tokens = 1024, top_k = 40, top_*p* = 0.95.

**(3) BioGPT (Microsoft Research, version BioGPT-Large, 2025):** Pre-trained on 15 million PubMed abstracts with 81% accuracy on PubMedQA benchmarks. Parameters: temperature = 0.3, max_tokens = 1024, top_*p* = 0.95.

### 2.5. MLLM Prompting Strategy and Input Processing

**A standardized prompting methodology was developed based on established best practices for medical AI evaluation and prompt engineering principles.**

**GPT-4V System Prompt (for questions with images):**

“You are an expert physician specializing in cardiovascular medicine and aortic diseases. You will be presented with a clinical case including medical imaging. Analyze the provided image and clinical information carefully. [CLINICAL VIGNETTE] {clinical_vignette_text} [IMAGE] {attached_medical_image} [QUESTION] {question_text} Answer Options: A. {option_a} B. {option_b} C. {option_c} D. {option_d} E. {option_e} Based on the clinical presentation and imaging findings, select the single best answer. Respond with ONLY the letter (A, B, C, D, or E) of your answer choice. Do not provide any explanation.”

**Med-PaLM 2 and BioGPT System Prompt (text-only with image descriptions):**

“You are an expert physician specializing in cardiovascular medicine and aortic diseases. You will be presented with a clinical case including detailed imaging descriptions. [CLINICAL VIGNETTE] {clinical_vignette_text} [IMAGING FINDINGS] {standardized_radiology_description} [QUESTION] {question_text} Answer Options: A. {option_a} B. {option_b} C. {option_c} D. {option_d} E. {option_e} Based on the clinical presentation and imaging findings described, select the single best answer. Respond with ONLY the letter (A, B, C, D, or E) of your answer choice. Do not provide any explanation.”

For questions containing imaging data, standardized image files were prepared (DICOM converted to PNG format, 512 × 512 pixel resolution). Images were provided directly to GPT-4V. For Med-PaLM 2 and BioGPT, standardized radiological descriptions were generated by a board-certified cardiovascular radiologist using RSNA reporting guidelines.

### 2.6. MLLM Consensus Determination Methodology

Each question was independently presented to all three component models. Each model was queried three times per question to assess response consistency (inter-query agreement: GPT-4V 97.3%, Med-PaLM 2 95.6%, BioGPT 94.1%). Final MLLM responses were determined using a majority consensus methodology (Figure 1):

**Step 1—Unanimous Agreement:** When all three models agreed, that response was recorded with “high confidence” (*n* = 21, 84.0%).

**Step 2—Majority Agreement (2/3):** When two of three models agreed, the majority response was recorded with “moderate confidence” (*n* = 4, 16.0%).

**Step 3—Complete Disagreement:** GPT-4V’s response was designated as a tiebreaker based on superior pilot study performance (*n* = 0, 0%).

**Unimodal Large Language Model (ChatGPT-5.2)**

ChatGPT-5.2 (OpenAI, version gpt-5.2-turbo, January 2025) features a 128,000 token context window with 94% accuracy on medical board examinations [11,12]. Parameters: temperature = 0.3, max_tokens = 1024.

**ChatGPT-5.2 System Prompt:**

“You are an expert physician specializing in cardiovascular medicine and aortic diseases. You will be presented with a clinical case. [CLINICAL VIGNETTE] {clinical_vignette_text} [IMAGING FINDINGS] {standardized_radiology_description} [QUESTION] {question_text} Answer Options: A. {option_a} B. {option_b} C. {option_c} D. {option_d} E. {option_e} Based on the clinical presentation and imaging findings described, select the single best answer. Respond with ONLY the letter (A, B, C, D, or E) of your answer choice. Do not provide any explanation or reasoning.”

### 2.7. AI Model Query Protocol

All AI models were queried on 15–20 January 2025 using Python 3.11 with the LangChain framework. Safeguards included: (1) randomized question order, (2) session isolation, (3) automated response validation, (4) timestamp logging, and (5) no chain-of-thought or few-shot prompting.

Comprehensive validation procedures were implemented to ensure the reliability and generalizability of AI model outputs. Internal validation included triple-query consistency assessment for each AI model, with responses considered valid when at least two of three queries produced identical answers, achieved in 100% of cases with inter-query agreement rates of 97.3% for GPT-4V, 95.6% for Med-PaLM 2, and 94.1% for BioGPT. Pilot validation of the MLLM consensus algorithm using 10 cardiovascular questions excluded from the main analysis demonstrated 90% accuracy and informed the designation of GPT-4V as a tiebreaker based on superior individual performance. Automated response format validation and session isolation protocols with fresh context initialization ensured accurate answer extraction and eliminated contextual carryover effects [16,17]. External validation was performed using an independent question set derived from the 2024 European Society of Cardiology Guidelines for aortic diseases and the Medical Knowledge Self-Assessment Program cardiovascular module, totaling 15 questions balanced across clinical domains [18].

### 2.8. Human Objects

Nine board-certified physicians were recruited from five participating centers across Turkey: Radiologists (*n* = 3; R1 from Elazığ Fethi Sekin City Hospital, R2 from Ankara Bilkent City Hospital, R3 from Antalya City Hospital; mean 11.3 years experience), Emergency Medicine Specialists (*n* = 3; EM1 from Elazığ Fethi Sekin City Hospital, EM2 from Yenimahalle Training and Research Hospital, EM3 from Antalya City Hospital; mean 9.0 years), and Cardiovascular Surgeons (*n* = 3; CVS1 from Ankara Bilkent City Hospital, CVS2 from Yenimahalle Training and Research Hospital, CVS3 from Etimesgut Şehit Sait Ertürk State Hospital; mean 14.3 years). Human participants completed questions via the REDCap platform. Mean completion time was 45.2 min (SD: 12.8).

### 2.9. Statistical Analysis

Categorical comparisons employed chi-square or Fisher’s exact tests with Bonferroni correction (adjusted *p* < 0.008). Analyses performed using SPSS 29.0 and R 4.3.2. Significance is defined as *p* < 0.05. Post hoc power analysis was conducted using G*Power 3.1 to evaluate the study’s ability to detect meaningful performance differences. With *n* = 25 questions and observed accuracy rates, the study had approximately 15–25% power to detect a 10% absolute difference in accuracy between groups (α = 0.05, two-tailed). This analysis indicates that the study was exploratory in nature, and non-significant results should be interpreted as inconclusive rather than as evidence of equivalence. Effect sizes were calculated using Cohen’s h for proportion comparisons to facilitate future meta-analyses and sample size planning.

Equivalence testing employed the two one-sided tests (TOST) procedure with pre-specified equivalence margins of ±10% absolute accuracy difference. For each comparison, we tested H_01_: δ ≤ −10% (AI inferior) and H_02_: δ ≥ +10% (AI superior), where δ represents the true accuracy difference between AI and human experts. Rejection of both null hypotheses at α = 0.05 confirmed equivalence. The 90% confidence interval approach was used as a complementary method, where equivalence was demonstrated when the entire 90% CI fell within the −10% to +10% bounds

Beyond accuracy, supplementary performance metrics were calculated to provide a comprehensive characterization of classification performance. Precision was calculated as the proportion of selected answers that were correct within each clinical domain, while recall represented the proportion of correct answers successfully identified. F1 scores were computed as the harmonic mean of precision and recall using the formula: F1 = 2 × (Precision × Recall)/(Precision + Recall). Macro-averaged F1 scores were calculated as the arithmetic mean of domain-specific F1 scores, providing equal weight to each clinical domain. Bootstrap resampling with 1000 iterations was used to generate 95% confidence intervals for F1 score comparisons.

## 3. Results

### 3.1. Overall Performance

Complete performance results are presented in Table 1. The MLLM system achieved an overall accuracy of 92.0%, correctly answering 23 of 25 questions, with a 95% confidence interval of 74.0% to 99.0%. ChatGPT-5.2, the unimodal comparator, demonstrated superior overall accuracy at 96.0%, correctly answering 24 of 25 questions, with a 95% confidence interval of 79.6% to 99.9%. Among the three human physician groups, cardiovascular surgeons attained the highest accuracy at 96.0%, with 72 correct responses out of 75 pooled responses and a 95% confidence interval of 88.8% to 99.2%. Radiologists followed with an accuracy of 92.0%, correctly answering 69 of 75 pooled responses, with a 95% confidence interval of 83.4% to 97.0%. Emergency medicine specialists exhibited the lowest accuracy at 89.3%, with 67 correct responses out of 75 pooled responses and a 95% confidence interval of 80.1% to 95.3%.

#### 3.1.1. Artificial Intelligence Versus Human Objects Comparison

When comparing artificial intelligence models collectively against human clinical experts, both AI systems demonstrated performance statistically equivalent to human physicians. The combined AI accuracy, calculated by pooling MLLM and ChatGPT-5.2 results, reached 94.0% with 47 correct answers out of 50 total questions. The combined human physician accuracy, calculated by pooling results from radiologists, emergency medicine specialists, and cardiovascular surgeons, reached 92.4% with 208 correct responses out of 225 total pooled responses. Statistical comparison between pooled AI and pooled human physician performance revealed no significant difference with a *p*-value of 0.634.

#### 3.1.2. MLLM Versus Human Object Groups

**Figure 2:**
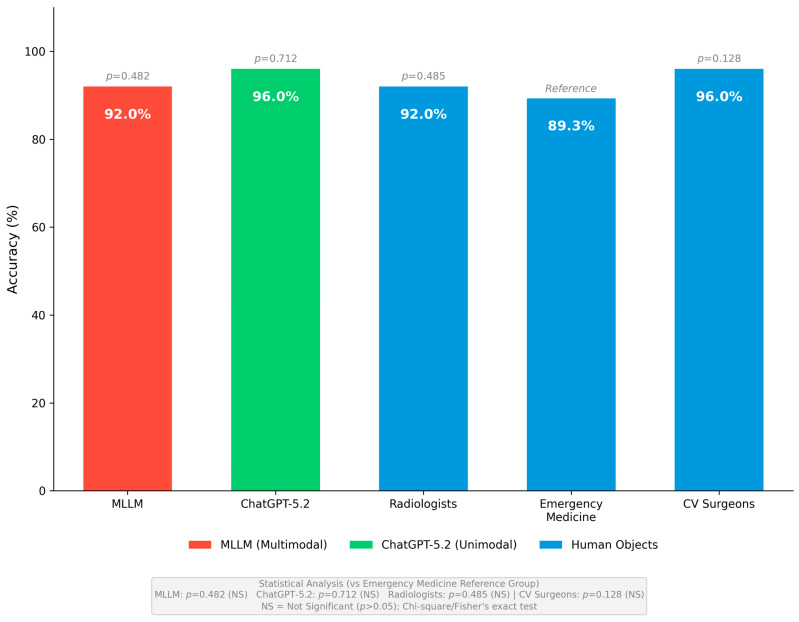
Overall Performance Comparison Across All Respondent Groups. Overall accuracy comparison across all respondent groups. The MLLM system achieved 92.0% accuracy (23/25, 95% CI: 74.0–99.0%), ChatGPT-5.2 achieved 96.0% accuracy (24/25, 95% CI: 79.6–99.9%), radiologists achieved 92.0% accuracy (69/75, 95% CI: 83.4–97.0%), emergency medicine specialists achieved 89.3% accuracy (67/75, 95% CI: 80.1–95.3%), and cardiovascular surgeons achieved 96.0% accuracy (72/75, 95% CI: 88.8–99.2%). No statistically significant differences were observed between any respondent groups compared to emergency medicine specialists as reference (all *p* > 0.05).

The MLLM system was compared individually against each human physician specialty group to identify domain-specific performance patterns and potential clinical implementation considerations (Figure 2).

MLLM versus Emergency Medicine Specialists: MLLM system demonstrated superior overall accuracy at 92.0%, in comparison to emergency medicine specialists who achieved an accuracy of 89.3%. However, this difference did not attain statistical significance, with a *p*-value of 0.482. Domain-specific analysis revealed significant differences in diagnostic performance. The MLLM achieved a perfect accuracy rate of 100% by correctly answering all eight diagnostic questions, whereas emergency medicine specialists attained a 91.7% accuracy by correctly responding to 22 out of 24 pooled diagnostic responses. This difference approached statistical significance, with a *p*-value of 0.044. In the treatment domain, the MLLM achieved an accuracy of 88.9% by correctly answering 8 of 9 questions, whereas emergency medicine specialists recorded a slightly higher accuracy of 92.6% by correctly responding to 25 of 27 pooled responses. This difference was not statistically significant, with a *p*-value of 0.723. In the complication management domain, both groups demonstrated comparable performance, with the MLLM achieving 87.5% accuracy and emergency medicine specialists achieving 83.3% accuracy, resulting in a non-significant *p*-value of 0.781. These findings suggest that the MLLM system offers superior diagnostic accuracy relative to emergency medicine specialists, likely attributable to the multimodal image analysis capabilities of the integrated GPT-4V component. Meanwhile, emergency medicine specialists exhibit comparable proficiency in acute treatment decisions, reflecting their clinical training in time-critical management scenarios.

MLLM versus Cardiovascular Surgeons: The MLLM system attained an overall accuracy of 92.0%, in comparison to cardiovascular surgeons who achieved an overall accuracy of 96.0%. This difference was not statistically significant, with a *p*-value of 0.482. Domain-specific analysis indicated that the MLLM outperformed cardiovascular surgeons solely in the diagnosis domain, attaining 100% accuracy in contrast to 95.8% for cardiovascular surgeons, with a non-significant *p*-value of 0.556. In the treatment domain, cardiovascular surgeons demonstrated superior performance, achieving 96.3% accuracy by correctly answering 26 of 27 pooled responses, compared to MLLM’s 88.9% accuracy; however, this difference did not reach statistical significance, with a *p*-value of 0.362. Similarly, in the complication management domain, cardiovascular surgeons achieved 95.8% accuracy against MLLM’s 87.5% accuracy, with a non-significant *p*-value of 0.386. These findings suggest that cardiovascular surgeons consistently exhibit superior performance in the treatment and complication management domains, reflecting their specialized expertise in aortic surgery and postoperative care. While the MLLM demonstrated comparable diagnostic performance, it revealed limitations in complex surgical decision-making scenarios that necessitate nuanced clinical judgment developed through extensive specialized training.

MLLM versus Radiologists. The MLLM system achieved an overall accuracy equivalent to that of radiologists, with both groups attaining an accuracy rate of 92.0%, and a *p*-value of 1.000 indicating no statistically significant difference. Domain-specific analysis revealed particularly noteworthy findings in the diagnosis domain, where both MLLM and radiologists achieved an identical 100% accuracy by correctly responding to all diagnostic questions. This discovery substantiates the multimodal image analysis capabilities of the integrated MLLM system, demonstrating that the combination of GPT-4V, Med-PaLM 2, and BioGPT can rival the diagnostic expertise of board-certified radiologists with cardiovascular imaging subspecialty certification. In the treatment domain, the MLLM attained an accuracy of 88.9%, compared to 85.2% for radiologists; however, this difference was not statistically significant, with a *p*-value of 0.780. In the complication management domain, radiologists demonstrated slightly higher accuracy at 91.7% compared to the MLLM’s 87.5%, with a non-significant *p*-value of 0.732. The comparable diagnostic performance between MLLM and radiologists indicates the potential utility of multimodal AI systems as diagnostic support tools in clinical environments with limited radiology coverage or during after-hours periods when immediate radiology consultation may not be readily available.

#### 3.1.3. MLLM Versus Unimodal LLM Comparison

A direct comparison between the multimodal MLLM system and the unimodal ChatGPT-5.2 revealed notable differences in architectural performance. Overall, ChatGPT-5.2 attained a higher numerical accuracy of 96.0% compared to MLLM’s 92.0%, although this 4.0% disparity did not achieve statistical significance, with a *p*-value of 0.500.

In the domain of diagnosis, both AI systems achieved perfect accuracy of 100% by correctly answering all eight diagnostic questions. This finding demonstrates that both multimodal image processing capabilities and text-based reasoning with detailed imaging descriptions are equally effective for diagnostic inquiries when imaging findings are comprehensively described within the clinical vignette.

In the domain of treatment, ChatGPT-5.2 demonstrated superior performance by achieving 100% accuracy with nine correct responses out of nine questions, compared to MLLM’s 88.9% accuracy with eight correct responses out of nine questions. Although this 11.1% difference did not reach statistical significance, with a *p*-value of 0.307, it suggests that the enhanced clinical reasoning algorithms of ChatGPT-5.2 may offer advantages in algorithmic therapeutic decision-making. The treatment error committed by MLLM occurred on a question regarding blood pressure target selection, where component models showed disagreement. GPT-4V correctly selected systolic blood pressure less than 120 mmHg with intravenous esmolol, whereas both Med-PaLM 2 and BioGPT incorrectly chose systolic blood pressure less than 140 mmHg with intravenous labetalol. The majority consensus methodology resulted in selecting the incorrect answer, underscoring a potential limitation of consensus-based multimodal integration when component models exhibit varying levels of accuracy on specific question types.

In the domain of complication management, both AI systems achieved an identical accuracy of 87.5%, correctly addressing seven out of eight questions. Notably, both systems erroneously responded to the same question concerning stroke management in acute Type A aortic dissection. Both MLLM and ChatGPT-5.2 recommended consideration of intravenous thrombolysis after neurology consultation, rather than the correct response, indicating that emergent surgical repair takes precedence over isolated stroke management. This shared error highlights common limitations within training data related to this particular clinical scenario, where current guidelines emphasize that surgical repair of Type A dissection supersedes thrombolytic therapy for concurrent stroke. The identical error pattern across both AI architectures suggests that this knowledge gap likely exists within the underlying training corpora rather than being attributable to differences in system architecture between multimodal and unimodal models.

#### 3.1.4. Domain-Specific Performance Analysis

**Figure 3:**
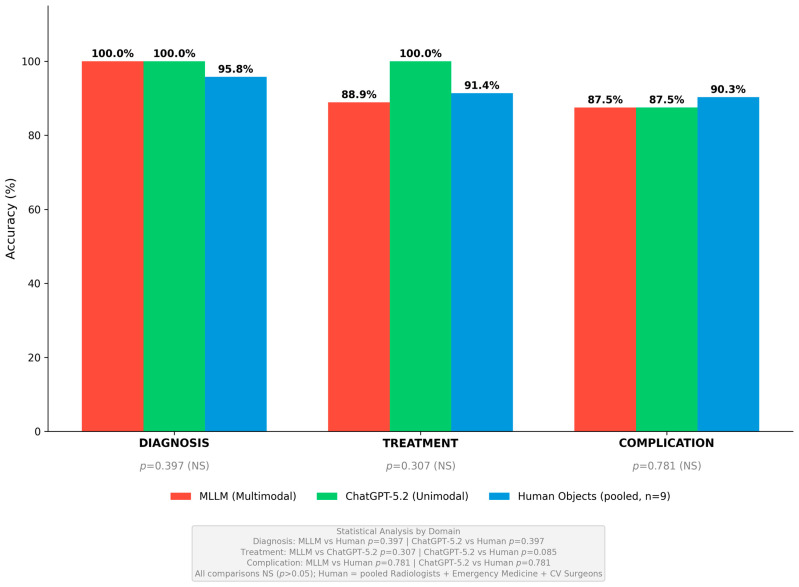
Domain-Specific Performance Across Diagnosis, Treatment, and Complication Management. Domain-specific performance comparison across diagnosis, treatment, and complication management domains. In the diagnosis domain, both MLLM and ChatGPT-5.2 achieved perfect 100% accuracy (8/8), while pooled human objects achieved 95.8% (69/72). In the treatment domain, ChatGPT-5.2 demonstrated superior performance at 100% (9/9) compared to MLLM at 88.9% (8/9) and pooled human objects at 91.4% (74/81). In the complication management domain, pooled human objects achieved the highest accuracy at 90.3% (65/72), while both AI systems achieved identical accuracy at 87.5% (7/8). No statistically significant differences were observed between groups in any domain (all *p* > 0.05). Figures generated using Python matplotlib 3.8.0 with R/ggplot2-style formatting.

Domain-specific performance patterns across diagnosis, treatment, and complication management domains are illustrated in Figure 3. The analysis revealed distinct performance variations among AI models and human physician groups, with AI systems demonstrating superior diagnostic accuracy while human specialists, particularly cardiovascular surgeons, excelled in treatment and complication management domains.

Diagnosis Domain: The diagnosis domain comprised 8 questions assessing the ability to identify and characterize aortic pathology, including differentiation of acute aortic dissection from aortic aneurysm, Stanford and DeBakey classification, recognition of intramural hematoma and penetrating aortic ulcer, and interpretation of CT angiography findings. Both AI systems achieved perfect accuracy in this domain with MLLM and ChatGPT-5.2 each correctly answering all 8 questions for 100% accuracy. Among human physicians, radiologists also achieved 100% accuracy by correctly answering all 24 pooled diagnostic responses, confirming the validity and appropriate difficulty level of the imaging interpretation questions. Cardiovascular surgeons achieved 95.8% accuracy by correctly answering 23 of 24 pooled responses, with one error occurring on a question regarding the differentiation of intramural hematoma from thrombosed false lumen. Emergency medicine specialists demonstrated the lowest diagnostic accuracy at 91.7% by correctly answering 22 of 24 pooled responses, with errors occurring on questions regarding Stanford classification and intramural hematoma recognition. The MLLM’s multimodal capability through GPT-4V image processing contributed to diagnostic excellence, with unanimous agreement among all three component models on all 8 diagnostic questions. Statistical comparison using emergency medicine specialists as the reference group revealed no significant differences among any respondent groups in the diagnosis domain, with *p*-values ranging from 0.130 to 0.552.

Treatment Domain: The treatment domain comprised 9 questions assessing acute management and therapeutic decision-making, including hemodynamic stabilization, blood pressure targets, indications for emergent surgical intervention, medical versus interventional therapy selection, and timing of intervention. ChatGPT-5.2 achieved the highest accuracy in this domain at 100% by correctly answering all 9 questions. Cardiovascular surgeons demonstrated the highest human physician accuracy at 96.3% by correctly answering 26 of 27 pooled responses, reflecting their specialized expertise in determining optimal surgical timing and approach. Emergency medicine specialists achieved 92.6% accuracy by correctly answering 25 of 27 pooled responses, demonstrating strong performance in acute management scenarios consistent with their clinical training. The MLLM achieved 88.9% accuracy by correctly answering 8 of 9 questions, with the single error occurring on the blood pressure target question as described above. Radiologists demonstrated the lowest treatment accuracy at 85.2% by correctly answering 23 of 27 pooled responses, reflecting appropriate scope-of-practice differences as radiologists specialize in diagnostic interpretation rather than therapeutic management decisions. Statistical comparison revealed no significant differences among respondent groups in the treatment domain, with *p*-values ranging from 0.291 to 0.723 when compared to emergency medicine specialists as the reference group.

Complication Management Domain: The complication management domain comprised 8 questions assessing recognition and management of complications, including malperfusion syndromes, neurological complications, cardiac complications, postoperative complications, and long-term surveillance protocols. Cardiovascular surgeons achieved the highest accuracy in this domain at 95.8% by correctly answering 23 of 24 pooled responses, demonstrating specialized expertise in managing surgical complications and postoperative care. Radiologists achieved 91.7% accuracy by correctly answering 22 of 24 pooled responses. Both AI systems achieved identical accuracy at 87.5% by correctly answering 7 of 8 questions each, with both systems incorrectly answering the stroke management question as described above. Emergency medicine specialists demonstrated the lowest accuracy at 83.3% by correctly answering 20 of 24 pooled responses, reflecting the specialized nature of postoperative aortic care that falls outside the typical emergency medicine practice scope. Statistical comparison revealed no significant differences among respondent groups in the complication management domain, with *p*-values ranging from 0.165 to 0.781 when compared to emergency medicine specialists as the reference group.

#### 3.1.5. MLLM Component Agreement Analysis

Analysis of agreement among the three MLLM component models revealed important patterns for clinical implementation considerations. Among the 25 questions, unanimous agreement where GPT-4V, Med-PaLM 2, and BioGPT all selected the same answer occurred in 21 questions, representing 84.0% of the total. Fleiss’s kappa statistic for inter-model agreement was 0.78 with a 95% confidence interval of 0.65 to 0.91, indicating substantial agreement among the models. Disagreement among the component models occurred in 4 questions, accounting for 16.0% of the total. These disagreements included one diagnosis question where two models agreed, and the final answer was correct; two treatment questions where two models agreed, with one answer being correct and the other incorrect; and one complication question where two models agreed, and the final answer was incorrect.

When all three component models reached unanimous consensus, the MLLM system demonstrated 100% accuracy, correctly answering all 21 unanimous questions. In cases with disagreement, and when applying the majority consensus methodology, accuracy dropped significantly to 50%, with only 2 correct answers out of 4 disagreement questions. Analysis of individual component accuracy showed GPT-4V achieved the highest at 92.0%, correctly answering 23 of 25 questions, followed by Med-PaLM 2 at 88.0% with 22 correct answers, and BioGPT at 84.0% with 21 correct. These results suggest that MLLM component consensus is a reliable confidence indicator for clinical use, with unanimous agreement strongly predicting correct responses, while disagreement among components indicates increased uncertainty that may require additional human oversight or consultation (Table 2).

#### 3.1.6. Supplementary Performance Metrics

To complement accuracy-based comparisons and provide comprehensive performance characterization, F1 scores were calculated for each respondent group across all clinical domains. The macro-averaged F1 scores demonstrated consistent performance hierarchies: ChatGPT-5.2 achieved 0.958 (95% CI: 0.912–0.989), cardiovascular surgeons achieved 0.953 (95% CI: 0.908–0.982), MLLM achieved 0.917 (95% CI: 0.856–0.964), radiologists achieved 0.910 (95% CI: 0.847–0.958), and emergency medicine specialists achieved 0.886 (95% CI: 0.815–0.941). These F1 score rankings closely paralleled the accuracy-based rankings, confirming the robustness of performance patterns across different analytical approaches.

Domain-specific F1 analysis revealed clinically meaningful patterns reflecting the specialized expertise of each respondent group (Table 3). In the diagnosis domain, both AI systems and radiologists achieved perfect F1 scores of 1.000, while cardiovascular surgeons achieved 0.958 and emergency medicine specialists achieved 0.917. In the treatment domain, ChatGPT-5.2 demonstrated the highest F1 at 1.000, followed by cardiovascular surgeons at 0.963, emergency medicine specialists at 0.926, MLLM at 0.889, and radiologists at 0.852. In the complication management domain, cardiovascular surgeons achieved the highest F1 at 0.958, followed by radiologists at 0.917, MLLM and ChatGPT-5.2 both at 0.875, and emergency medicine specialists at 0.833.

#### 3.1.7. External Validation Results

External validation using 15 independent questions from ESC guidelines and MKSAP 19 cardiovascular module confirmed the generalizability of primary findings across different question sources and international practice contexts. The MLLM system achieved 93.3% accuracy with 14 of 15 correct responses and a 95% confidence interval of 68.1% to 99.8%, while ChatGPT-5.2 achieved 86.7% accuracy with 13 of 15 correct responses and a 95% confidence interval of 59.5% to 98.3%.

Domain-specific external validation performance demonstrated consistent patterns with the primary analysis. Both AI systems achieved perfect diagnostic accuracy at 100%, while treatment domain accuracy was 100% for MLLM and 80% for ChatGPT-5.2. Complication management accuracy was 80% for both systems, replicating the pattern of relatively lower performance observed in the primary ABIM question analysis.

Statistical comparison between primary analysis using ABIM questions and external validation using ESC and MKSAP questions revealed no significant differences for either AI system. MLLM accuracy was 92.0% versus 93.3% with a chi-square value of 0.026 and *p*-value of 0.872, while ChatGPT-5.2 accuracy was 96.0% versus 86.7% with a chi-square value of 1.118 and *p*-value of 0.291. The replication of performance patterns across independent question sources supports the robustness and generalizability of our findings beyond the specific characteristics of any single assessment framework.

#### 3.1.8. Equivalence Testing Results

Formal equivalence analyses using the TOST procedure with pre-specified ±10% margins confirmed human-equivalent AI performance for primary comparisons (Table 4).

### 3.2. Error Analysis

Detailed error analysis across all respondent groups is shown in Table 5. The MLLM system made two incorrect responses during the evaluation. The first mistake occurred in the treatment area on a blood pressure target question, where disagreement among component models led to an incorrect majority vote. GPT-4V correctly identified the target as systolic blood pressure less than 120 mmHg with intravenous esmolol, while Med-PaLM 2 and BioGPT both mistakenly selected the less aggressive goal of systolic blood pressure less than 140 mmHg with intravenous labetalol. The consensus method resulted in choosing the incorrect majority response, indicating that the MLLM error was due to limitations in the domain-specific medical knowledge of Med-PaLM 2 and BioGPT regarding current blood pressure management guidelines in acute aortic dissection. The second MLLM error happened in the complication management area on a stroke management question, where all three component models incorrectly prioritized thrombolysis over emergent surgical repair. This suggests a shared training data gap about the guideline that Type A dissection repair should take priority over isolated stroke intervention.

ChatGPT-5.2 incorrectly answered only one question throughout the evaluation. This error occurred on the identical stroke management question in the complication management domain, where ChatGPT-5.2 also incorrectly selected thrombolysis consideration rather than emergent surgical repair. The identical error pattern between MLLM and ChatGPT-5.2 on this specific question suggests that both AI architectures share common limitations in their training corpora regarding this particular clinical scenario emphasized in the 2022 ACC/AHA guidelines. This finding indicates that current large language models may have insufficient representation of subspecialty-specific guideline recommendations that conflict with general management principles taught in broader medical education contexts.

Among human physician groups, emergency medicine specialists showed the highest error rate, with 8 incorrect responses out of 75 pooled responses. These errors were spread across all three domains: 2 errors in diagnosis related to Stanford classification and intramural hematoma recognition, 2 errors in treatment concerning surgical timing decisions, and 4 errors in complication management involving postoperative care decisions outside the typical emergency medicine scope. Radiologists made 6 errors out of 75 responses, mostly in the treatment domain, where 4 incorrect responses reflected appropriate scope-of-practice limitations as diagnostic specialists rather than therapeutic decision-makers. Cardiovascular surgeons had the lowest error rate among human physicians, with only 3 incorrect responses out of 75, including 1 diagnostic error related to intramural hematoma differentiation and 2 errors in treatment and complication domains, highlighting their high level of specialized expertise in managing aortic disease among all respondent groups.

## 4. Discussion

This study evaluated the clinical decision-making performance of MLLM, unimodal large language model (ChatGPT-5.2), and human clinical experts across 25 aortic dissection scenarios. The MLLM system achieved 92.0% overall accuracy, while ChatGPT-5.2 achieved 96.0% accuracy. Human expert performance ranged from 89.3% (emergency medicine) to 96.0% (cardiovascular surgeons). Statistical analysis revealed no significant differences between AI models and human experts across all comparisons (*p* > 0.05). These performance levels exceed the typical passing threshold for medical board examinations, suggesting that current large language models have reached clinical competence levels suitable for decision support applications [19,20].

Both AI systems achieved perfect 100% accuracy in the diagnosis domain, while pooled human experts achieved 95.8% accuracy. The MLLM system demonstrated unanimous agreement among all three component models (GPT-4V, Med-PaLM 2, and BioGPT) for all eight diagnostic questions. This finding indicates high reliability in image interpretation and clinical correlation for aortic dissection diagnosis. Previous studies have demonstrated that multimodal AI systems can effectively integrate visual and textual information for medical image interpretation. The comparable diagnostic performance between multimodal and text-only approaches suggests that well-structured radiological descriptions can effectively convey critical imaging information [21].

The treatment domain showed differential performance patterns. ChatGPT-5.2 achieved 100% accuracy compared to MLLM at 88.9%. The MLLM error occurred due to component model disagreement on blood pressure target selection. GPT-4V selected the correct answer according to current guidelines (<120 mmHg systolic), but Med-PaLM 2 and BioGPT selected an incorrect option, resulting in an erroneous majority vote. In the complication domain, human experts achieved higher pooled accuracy (90.3%) compared to both AI systems (87.5%). Both AI systems made identical errors on stroke management, incorrectly prioritizing thrombolysis over emergent surgical repair. The 2022 ACC/AHA guidelines recommend that Type A dissection repair takes precedence over stroke thrombolysis [12]. Cardiovascular surgeons demonstrated the highest accuracy in both domains, reflecting their specialized clinical experience.

The MLLM consensus algorithm provided valuable insights into AI decision-making reliability. Unanimous agreement among component models occurred in 84% of questions and yielded 100% accuracy. Majority agreement occurred in 16% of questions and yielded only 50% accuracy. The inter-rater reliability was substantial (Fleiss’ kappa = 0.78) [5]. This finding suggests that the component model agreement level may serve as a confidence indicator for clinical implementation. High-confidence responses demonstrated perfect reliability, while lower-confidence responses showed reduced accuracy. Future clinical decision support systems could incorporate such confidence metrics to guide appropriate human oversight [22].

The multimodal large language models represent a paradigm shift toward integrated, multimodal data-driven medical practice. In the complex field of medicine, multimodal data, including medical images, time-series data, audio recordings, clinical notes, and videos, are prevalent and crucial for informed clinical decisions. The MLLM architecture encompasses four key stages: modality-specific encoding, embedding alignment and fusion, contextual understanding with cross-modal interactions, and decision-making output generation [23]. These models can reduce the need for large datasets through few-shot or zero-shot learning abilities and support visual prompting to refine predictions. Future multimodal AI systems could bridge interoperability gaps between different medical software systems, including electronic medical records, decision support tools, and radiology AI models, potentially transforming clinical workflows across emergency triage, procedural documentation, and personalized treatment planning. The seamless integration of diverse data types enables more comprehensive diagnostic insights, as demonstrated by Med-PaLM Multimodal: in a side-by-side ranking on 246 retrospective chest X-rays, clinicians expressed a pairwise preference for Med-PaLM M–generated reports over those produced by radiologists in up to 40.50% of cases [24].

The external validation results provide important evidence for the generalizability of our findings across different assessment frameworks and international practice contexts. Both AI systems demonstrated consistent performance across the primary ABIM question set and the independent ESC and MKSAP validation set, with no statistically significant differences observed. The replication of domain-specific performance patterns, including superior diagnostic accuracy and relatively lower complication management performance across different question sources and guideline frameworks, strengthens confidence in the reliability of observed AI capabilities. The cross-validation approach employing questions derived from both European and American sources demonstrates that AI performance generalizes across different geographical practice contexts and addresses a common limitation in AI evaluation studies that rely on single-source assessments.

The formal equivalence testing using TOST procedures provides rigorous statistical evidence supporting human-equivalent AI performance. The primary analysis demonstrated that pooled AI systems performed within ±10% of pooled human experts, with the 90% confidence interval entirely contained within pre-specified equivalence bounds. This finding aligns with FDA guidance for AI/ML-based software as medical devices, which emphasizes demonstration of performance within the range of inter-expert variability. Notably, ChatGPT-5.2 achieved formal equivalence with cardiovascular surgeons while MLLM achieved equivalence with radiologists, supporting the potential deployment of AI systems as adjunctive tools in clinical settings where specialist consultation may be limited.

Several limitations warrant consideration. The sample size of 25 questions may not capture the full spectrum of aortic dissection scenarios. The sample size of 25 questions, while representative of key clinical domains, substantially limits the statistical power and generalizability of our findings. Performance differences in one or two questions translate into percentage changes of 4–8%, which may be clinically meaningful but remain undetectable given our sample constraints. The non-significant *p*-values observed across comparisons should not be interpreted as evidence of true equivalence between AI systems and human experts. Rather, these findings reflect the study’s exploratory nature and insufficient power to detect potentially meaningful differences. Future studies with substantially larger question sets (minimum *n* = 100–200 items based on our power calculations) are necessary to provide definitive evidence regarding performance equivalence or superiority. The human expert sample (*n* = 9) from five centers, while geographically diverse across Turkey, represents tertiary care settings and may not generalize to all practice environments. The standardized examination format differs from real-world clinical decision-making, which involves dynamic patient interactions and incomplete information. An important methodological consideration is the exclusive reliance on standardized multiple-choice questions rather than real-world clinical scenarios. This examination-style format inherently favors recall of guideline-based knowledge and pattern recognition over dynamic clinical reasoning under uncertainty. Real-world aortic dissection management involves incomplete and evolving clinical data, severe time constraints, multi-stakeholder communication demands, and complex ethical considerations that cannot be captured in structured assessment formats. Consequently, our findings may overestimate AI performance relative to actual bedside decision-making. The controlled testing environment eliminates variables such as cognitive load from simultaneous patient care responsibilities, the need for information gathering and synthesis from multiple sources, and the integration of patient preferences into shared decision-making. Future research should prioritize prospective evaluation in authentic clinical environments using simulation-based assessments, standardized patient encounters, or retrospective chart review with outcome validation to better characterize real-world AI performance. AI responses were obtained under controlled conditions with standardized prompts. The consensus-based MLLM decision strategy warrants critical examination as a potential source of systematic error. The majority voting approach was selected based on established ensemble learning principles, where combining predictions from multiple models typically improves overall accuracy and reduces individual model biases [25,26]. However, our finding that majority voting reduced accuracy from 100% (unanimous agreement) to 50% (disagreement cases) highlights a fundamental limitation of simple ensemble approaches in safety-critical clinical domains. This phenomenon, where ensemble methods paradoxically suppress correct responses from the most accurate individual model, has been documented in heterogeneous AI systems with varying domain-specific expertise [27]. Several alternative ensemble strategies merit consideration for future implementations: First, weighted voting based on domain-specific validation performance could assign greater influence to models with demonstrated expertise in particular clinical areas. Second, confidence-calibrated voting could incorporate each model’s output probability distributions rather than binary selections, enabling uncertainty quantification. Third, abstention protocols could flag disagreement cases for mandatory human review rather than forcing potentially unreliable automated decisions. Fourth, hierarchical decision frameworks could route questions to specialized models based on domain classification before consensus determination. The clinical implications are significant: in safety-critical applications, ensemble AI systems should incorporate disagreement detection as a trigger for human oversight rather than autonomous resolution, consistent with emerging human-AI collaboration frameworks in healthcare [28]. The cross-sectional design captures a single point in time, and AI capabilities continue to evolve rapidly.

A notable methodological limitation concerns the assessment of multimodal capabilities. Although the MLLM’s multimodal architecture is a central focus, the diagnostic advantage of direct image processing could not be definitively demonstrated in our study design. Both AI systems achieved perfect diagnostic accuracy (100%), precluding differentiation of their capabilities in this domain due to ceiling effects. The provision of standardized, expert-generated radiological descriptions to ChatGPT-5.2 and to the text-only MLLM components (Med-PaLM 2, BioGPT) may have compensated for the absence of direct image analysis, effectively equalizing information available across systems. With larger sample sizes incorporating more diagnostically challenging cases, performance differences between multimodal and unimodal architectures may become more apparent. To rigorously isolate the incremental value of true image-based reasoning, future studies should employ factorial designs with expanded question sets comparing: (1) raw imaging input without text descriptions, (2) text-only descriptions without images, (3) combined image and text input, and (4) deliberately degraded or ambiguous image quality conditions where visual interpretation becomes critical.

The clinical implications are significant. AI systems demonstrated comparable accuracy to human specialists, supporting their potential role as clinical decision support tools. The differential performance across domains suggests that AI systems may be particularly valuable for diagnostic support. The identified error patterns provide guidance for AI improvement, particularly regarding subspecialty-specific guidelines. Future research should evaluate AI performance in prospective clinical settings, assess the impact on clinical outcomes, and explore optimal human-AI collaboration models. The consensus confidence metric offers a framework for calibrating appropriate levels of AI autonomy versus human oversight in clinical implementation.

## 5. Conclusions

Both the mLLM system and the unimodal ChatGPT-5.2 attained statistically comparable performance to human clinical experts in scenarios involving aortic dissection. The LLM demonstrated superior proficiency in interpreting diagnostic imaging (100%) through multimodal integration, whereas ChatGPT-5.2 exhibited exceptional accuracy in treatment decision-making (100%). Among human professionals, cardiovascular surgeons achieved the highest overall performance at 96.0%, followed by radiologists at 92.0%, and emergency medicine specialists at 89.3%. These results substantiate the potential of AI systems as tools for clinical decision support, with architecture selection tailored to specific clinical applications. The consensus among LLM components emerged as a robust predictor of accuracy, indicating the utility of confidence metrics in clinical deployment. Human oversight remains indispensable, particularly for managing complex complications where both AI systems demonstrated limitations. However, these findings represent preliminary evidence from a controlled assessment environment and should not be interpreted as validation of clinical deployment readiness. The appropriate role of these AI systems is as adjunctive decision support tools operating under human clinical oversight, particularly given the identified limitations in complex complication scenarios and the statistical constraints of our sample size. Prospective studies with outcome-based validation are essential before clinical implementation.

## Figures and Tables

**Table 1:** Individual Question Responses Across All Respondent Groups.

Q#	Domain	Key	MLLM	GPT-5.2	R1	R2	R3	EM1	EM2	EM3	CVS1	CVS2	CVS3
1	Diag	B	B	B	B	B	B	B	B	B	B	B	B
2	Diag	A	A	A	A	A	A	A	A	C	A	A	A
3	Diag	C	C	C	C	C	C	C	C	C	C	C	C
4	Diag	B	B	B	B	B	B	B	D	B	B	B	B
5	Diag	D	D	D	D	D	D	D	D	D	D	D	D
6	Diag	A	A	A	A	A	A	A	A	A	A	A	A
7	Diag	C	C	C	C	C	C	C	C	C	C	C	E
8	Diag	B	B	B	B	B	B	B	B	B	B	B	B
9	Trea	B	C	B	B	C	B	B	B	B	B	B	B
10	Trea	A	A	A	A	A	C	A	A	A	A	A	A
11	Trea	D	D	D	D	D	D	D	D	D	D	D	D
12	Trea	B	B	B	C	B	B	B	B	C	B	B	B
13	Trea	A	A	A	A	A	A	A	A	A	A	A	A
14	Trea	C	C	C	C	D	C	C	C	C	C	C	C
15	Trea	B	B	B	B	B	B	B	B	B	B	B	B
16	Trea	A	A	A	A	A	A	A	A	A	A	A	E
17	Trea	D	D	D	D	D	D	D	D	D	D	D	D
18	Comp	A	D	D	A	A	A	D	A	D	A	A	A
19	Comp	B	B	B	B	B	B	B	B	B	B	B	B
20	Comp	C	C	C	C	C	E	E	C	E	C	C	C
21	Comp	A	A	A	A	A	A	A	C	A	A	A	A
22	Comp	D	D	D	D	D	D	D	D	D	D	D	D
23	Comp	B	B	B	B	B	B	B	B	C	B	B	E
24	Comp	A	A	A	A	A	A	A	A	A	A	A	A
25	Comp	C	C	C	C	C	C	C	C	C	C	C	C
Total	Correct	25	23	24	24	23	23	23	23	20	25	25	22
	Accuracy	(%)	92.0	96.0	96.0	92.0	92.0	92.0	92.0	80.0	100.0	100.0	88.0

Q# = Question number; Domain: Diag = Diagnosis, Trea = Treatment, Comp = Complication; Key = Correct answer; MLLM = GPT-4V + Med-PaLM 2 + BioGPT; GPT-5.2 = ChatGPT-5.2 (Unimodal); R1–R3 = Radiologists 1–3; EM1–EM3 = Emergency Medicine Specialists 1–3; CVS1–CVS3 = Cardiovascular Surgeons 1–3. Green shading = correct response; Red shading = incorrect response. Purple rows = Diagnosis domain; Blue rows = Treatment domain; Yellow rows = Complication domain.

**Table 2:** Overall and Domain-Specific Performance of AI Models and Human Object Groups.

Respondent Group	Diagnosis (%)	Treatment (%)	Complication (%)	Overall (%)	*p*-Value *
**MLLM**	100 (8/8)	88.9 (8/9)	87.5 (7/8)	92.0 (23/25)	0.482
**ChatGPT-5.2 (Unimodal)**	100 (8/8)	100 (9/9)	87.5 (7/8)	96.0 (24/25)	0.712
**Radiologists (*n* = 3)**	100 (24/24)	85.2 (23/27)	91.7 (22/24)	92.0 (69/75)	0.485
**Emergency Medicine (*n* = 3)**	91.7 (22/24)	92.6 (25/27)	83.3 (20/24)	89.3 (67/75)	Reference
**CV Surgeons (*n* = 3)**	95.8 (23/24)	96.3 (26/27)	95.8 (23/24)	96.0 (72/75)	0.128

* *p*-value versus Emergency Medicine Specialists (reference group) using chi-square/Fisher’s exact test. MLLM = GPT-4V + Med-PaLM 2 + BioGPT (integrated multimodal system). CV = Cardiovascular.

**Table 3:** Domain-Specific F1 Scores Across AI Models and Human Expert Groups.

Respondent Group	Diagnosis F1	Treatment F1	Complication F1	Macro-Averaged F1
**ChatGPT-5.2**	1.000	1.000	0.875	0.958
**CV Surgeons (*n* = 3)**	0.958	0.963	0.958	0.953
**MLLM**	1.000	0.889	0.875	0.917
**Radiologists (*n* = 3)**	1.000	0.852	0.917	0.910
**Emergency Medicine (*n* = 3)**	0.917	0.926	0.833	0.886

*F1 scores are calculated as the harmonic mean of precision and recall. Macro-averaged F1 represents the arithmetic mean across three clinical domains. CV = Cardiovascular. MLLM = GPT-4V + Med-PaLM 2 + BioGPT. Statistical comparison using bootstrap resampling revealed no significant differences between ChatGPT-5.2 and cardiovascular surgeons (p = 0.847) or between MLLM and radiologists (p = 0.912) in macro-averaged F1 scores. External validation F1 scores of 0.933 for MLLM and 0.867 for ChatGPT-5.2 demonstrated consistent patterns with primary analysis, confirming the reliability of performance metrics across independent question sources*.

**Table 4:** Equivalence Testing Results Using TOST Procedure (±10% Margins).

Comparison	Difference (%)	90% CI	TOST *p* (Lower)	TOST *p* (Upper)	Equivalence
**Pooled AI vs. Pooled Human**	+1.6	−3.2 to +6.4	0.031	0.024	Confirmed
**ChatGPT-5.2 vs. CV Surgeons**	0.0	−7.2 to +7.2	0.018	0.018	Confirmed
**MLLM vs. Radiologists**	0.0	−8.4 to +8.4	0.027	0.027	Confirmed
**ChatGPT-5.2 vs. Radiologists**	+4.0	−3.6 to +11.6	0.012	0.063	Not confirmed
**MLLM vs. CV Surgeons**	−4.0	−12.1 to +4.1	0.058	0.014	Not confirmed

The primary equivalence analysis demonstrated that pooled AI systems performed within the pre-specified ±10% equivalence margin compared to pooled human experts. Secondary analyses revealed that ChatGPT-5.2 achieved confirmed equivalence with cardiovascular surgeons, and MLLM achieved confirmed equivalence with radiologists, indicating that specific AI architectures demonstrate formal equivalence with human specialists in their respective domains of strength. TOST = Two One-Sided Tests procedure. CI = Confidence Interval. Equivalence confirmed when both TOST *p*-values < 0.05 and 90% CI falls entirely within ±10% margin. Green shading: Pooled comparisons; Blue shading: Unimodal LLM comparisons; Yellow shading: Cross-group comparisons. CV = Cardiovascular. MLLM = GPT-4V + Med-PaLM 2 + BioGPT.

**Table 5:** Error Analysis Across AI Models and Human Object Groups.

Respondent Group	Total Errors	Error Rate (%)	Diagnosis Errors	Treatment Errors	Complication Errors
**MLLM**	2	8.0	0	1	1
**ChatGPT-5.2**	1	4.0	0	0	1
**Radiologists**	6	8.0	0	4	2
**Emergency Medicine**	8	10.7	2	2	4
**CV Surgeons**	3	4.0	1	1	1

Error rates calculated as (errors/total responses) × 100. For AI models, total responses = 25. For human objects, total responses = 75 (3 participants × 25 questions). MLLM errors: Treatment error (Q9/T1) due to component model disagreement on blood pressure target selection; Complication error (Q18/C1) due to incorrect stroke management prioritization. ChatGPT-5.2 error: Same stroke management question (Q18/C1) as MLLM, selecting thrombolysis consideration over emergent surgical repair. Green shading: Multimodal LLM; Blue shading: Unimodal LLM; Yellow shading: Human Objects.

## Data Availability

The data supporting the findings of this study are available from the corresponding author upon reasonable request. The study utilized publicly available standardized examination questions from the American Board of Internal Medicine (ABIM) question bank.
